# Factors influencing patient satisfaction after treatments for early-stage non-small cell lung cancer

**DOI:** 10.1007/s00432-021-03795-0

**Published:** 2021-09-13

**Authors:** Cecilia Pompili, Sanjush Dalmia, Finn McLennan Battleday, Zoe Rogers, Kate Absolom, Hilary Bekker, Kevin Franks, Alex Brunelli, Galina Velikova

**Affiliations:** 1 Section of Patient Centred Outcomes Research, Leeds Institute for Medical Research at St James’s, University of Leeds, Leeds, UK; 2 School of Medicine, University of Leeds, Leeds, UK; 3 Leeds Institute of Health Sciences, University of Leeds, Worsley Building, Clarendon Way, Leeds, LS2 9NL UK; 4 Leeds Teaching Hospital, Leeds, UK; 5 Bexley Wing, St. James’ Institute of Oncology, Beckett Street, Leeds, LS9 7TF UK

**Keywords:** Quality of life, Lung cancer surgery, VATS, SABR, Patient-reported outcomes

## Abstract

**Purpose:**

Patient-reported outcome measures, including satisfaction with treatment decisions, provide important information in addition to clinical outcomes, survival and decision-making in lung cancer surgery. We investigated associations between preoperative clinical and socio-demographic factors and patient-reported satisfaction 6 weeks after radical treatment for early-stage non-small cell lung cancer (NSCLC).

**Methods:**

We conducted a sub-group analysis of the prospective observational longitudinal study of 225 participants in two treatment groups—surgical (VATS) and radiotherapy (SABR). The Patient Satisfaction Questionnaire-18 (PSQ-18) was used to measure patient satisfaction 6 weeks after treatment. Clinical variables, Index of Multiple Deprivation decile and Decision self-efficacy scores were used in regression analysis. Variables with a p level < 0.1 were used as independent predictors in generalised linear logistic regression analyses.

**Results:**

As expected, the two groups differed in pre-treatment clinical features. The SABR group experienced more grade 1–2 complications than the VATS group. No differences were found between the groups in any subscale of the PSQ-18 questionnaire. Patients experiencing complications or living in more deprived areas were more satisfied with care. Properative factors independently associated with patient satisfaction were the efficacy in decision-making and age.

**Conclusion:**

We showed that efficacy in treatment decision-making and age was the sole predictor of patient satisfaction with their care after radical treatment for early-stage NSCLC. Patients from more deprived areas and patients who suffered complications reported greater subsequent satisfaction. Involving patients in their care may improve satisfaction after treatment for early-stage NSCLC.

## Introduction

Lung cancer is associated with high morbidity and mortality. In addition, significant comorbidities often markedly reduce the patient’s baseline performance status. The high prevalence and significant symptomatic burden of lung cancer on patients is befitting to patient satisfaction as a surgical outcome measure.

UK National Institute for Clinical Excellence (NICE) guidelines recommend that early-stage non-small cell lung cancer is treated using video-assisted thoracoscopic surgery (VATS) for fit patients, and stereotactic ablation radiotherapy (SABR) for patients for whom VATS is not appropriate (NICE 2019).

Patient-reported outcome measures (PROMS) provide information additional to long-term survival, risk–benefit decision-making and surgical outcomes in lung cancer surgery. Patient satisfaction is one of many important PROMS and is vital to assess the quality of treatment (Chow et al. 2009). Patient satisfaction with treatment is a key quality indicator for lung cancer care (Brunelli and Rocco 2007) as it reflects a patient’s perception of the quality of care received. Although clinical indicators improve with surgery, it is likely that patient-reported experience of care gets worse after treatment due to the prevalence and impact of symptoms that result from different treatments. As a consequence, measuring patient perception of satisfaction with the process of care is essential to delivering effective practice and enhancing patient-centred management.

There are no specific lung cancer questionnaires to assess patient satisfaction with care. Satisfaction is an abstract and multi-dimensional concept which is difficult to directly observe or measure; therefore, it should be evaluated using a variety of multi-item scales (Avery et al. 2006). Measuring patient satisfaction requires validated tools, of which there are multiple options. Nevertheless, the consistent association of socio-economic inequality in relation to lung cancer incidence and mortality (Mackenback et al. 2004) is questioning if health disparity and challenges in access to lung cancer care can affect patient satisfaction after treatment.

The aim of this study is to investigate whether any specific preoperative factors, either clinical, demographic or patient-reported, were related to patient satisfaction with care at 6 weeks after surgical or stereotactic radiotherapy treatment of lung cancer.

We hypothesized that there is an association between patient decision-making efficacy for lung cancer treatment and their post-treatment satisfaction.

## Materials and methods

This study is a sub-analysis of the Lilac study, a prospective observational longitudinal study with repeated patient-reported outcome measures at baseline, 6 weeks and 3, 6 and 12 months after radical treatments for early-stage (stages I–II) non-small-cell lung cancer (NSCLC). The study cohort comprised consecutive patients undergoing VATS anatomical lung resection or SABR for early-stage NSCLC at a single UK centre during a period of 12 months. All participants signed a written consent form and the study received ethical approval from The National Research Ethics Service Yorkshire and the Humber-Leeds East Committee (REC Ref: 16/YH/0407). This study has been registered in the Clinical Trial database (ClinicalTrials.gov Identifier: NCT02882750).

### Treatment groups

The study population were patients with early-stage lung cancer treated with either VATS or SABR at Leeds Teaching Hospitals Trust over a period of 12 months. Patient eligibility for the study was assessed using the criteria listed in Table 1.

**Table 1:** Inclusion and exclusion criteria

Inclusion criteria	Exclusion criteria
Age 18 years and over	Advanced disease (III–IV stages)
Diagnosis of NSCLC either from histology or multi-disciplinary team meeting (MDT/Tumour Board) with agreement on > 95% likelihood of diagnosis based on radiological evidence or both	Patient included in another quality of life study, which may increase patient burden and bias the answers of the questionnaires
Decision for either surgery or SABR	
Able to give informed consent	
Able to understand the language of the questionnaire	

### Patient-reported outcomes questionnaires

Patients were invited to self-report patient-reported outcomes (PROs) using online secure access via QTool software at home or in clinic before the treatment and at follow-up intervals. Appropriate training for the use of the QTool was offered to both clinicians and patients (Holch et al. 2017). Paper administration was offered to patients without internet access. Clinicians with access to the electronic patient medical record (EPR) were able to consult the PROs in real time during consultations. Patients were collecting Quality of Life data through the administration of the European Organization for Research and Treatment (EORTC) Quality of Life Questionnaire (QLQ C-30) and its Lung Cancer specific Module (LC-13) at baseline, and post-treatment at 6 weeks, 3, 6 and 12 months. At the baseline, they were also collecting decision self-efficacy data and at 6 weeks patient satisfaction through the PSQ-18.

The Patient Satisfaction Questionnaire Short Form (PSQ-18) was chosen as it is a cross-cultural validated survey for use in different settings (Thayaparan and Mahdi 2013). It is short, reducing patient burden of filling in multiple questionnaires and covers the most important aspects of hospital attendance. The PSQ-18 questionnaire is an 18-item self-administered survey including different scales reflecting the perceived level of satisfaction in relation to the care provided by doctors. The team behind this Likert scale questionnaire proposed seven dimensions of patient satisfaction directed toward their doctors. These are general satisfaction, technical quality, interpersonal manner, communication, financial aspects, time spent with doctor, and accessibility and convenience (Thayaparan and Mahdi 2013). Patient satisfaction was only assessed at the first post-treatment time point (6 weeks). All items are scored (1–5) so that high scores reflect satisfaction with medical care. This was done to maximize response rate, as suggested by Bredart et al. (2005). Moreover, assessing satisfaction close to hospital recovery could allow for a better distinction among elements of satisfaction and higher response variability (Kane et al. 1997).

The Decision Self-Efficacy (DSE) scale is an 11-item questionnaire assessing the efficacy of decision-making with a 5-point response scale to generate a score between 0 and 100, with 0 indicating very low decision self-efficacy and 100 indicating very high decision self-efficacy. This instrument was shown to be reliable and valid for use in this population (Pompili et al. 2020). We administered the DSE at the pre-treatment time point.

### Analysis

We compared patient satisfaction measured using the PSQ-18 questionnaire between the VATS and SABR groups. Descriptive data summarise the characteristics of patients, and questionnaire responses.

We also explored preoperative factors that may predict patient satisfaction. The following variables were initially used in the analysis: age, preoperative forced expiratory volume in 1 s (FEV1%), gender, decision self-efficacy scale score (DSE), Index of multiple deprivation decile (IMD), Eastern Cooperative Oncology Group (ECOG) Performance Status (PS) and Carlson comorbidity index (CCI). Although the VATS and SABR groups were highly heterogeneous, limited numbers in each group meant it was not possible to run separate multivariable regression analyses for each group. One multivariable regression analysis of all 134 patients was carried out and adjusted for treatment type. Variables with a *p* value < 0.1 were used as independent predictors in generalised linear regression analysis.

## Results

### Recruitment

In total, 356 patients were eligible to participate in the study in a 12 months period, with 244 consenting to take part (60%). Of these, nineteen patients were excluded from the analysis: seven did not receive any treatments for oncological reasons, and twelve were deemed ineligible prior to treatment due to their suitability for open operation. Of the remaining 225 patients, 44 did not complete the trial: 23 died (13 in the SABR group and 10 in the VATS group); 9 became ineligible and 12 (eight patients in the SABR group and four in the VATS group) actively withdrew. Of the 12 patients who actively withdrew, five felt they had too many other time commitments to continue participation and the remaining seven did not provide a reason. Of the 225 participants, 91 failed to complete a patient satisfaction questionnaire at 6 weeks and thus were excluded from the analysis.

### Patient baseline demographics and clinical characteristics

The clinical details and baseline (pre-treatment) demographics of the 225 patients who underwent VATS or SABR are presented in Table 2. The two groups differ considerably in their pre-treatment clinical features. There were no major differences in the available demographic data for the total patients treated in the same period.

**Table 2:** Patient demographic and clinical details according to treatment

	SABR (*n* = 95) Mean or count	Median	IQR	Surgery (*n* = 130) Mean or count	Median	IQR	*p* value
Age	74.3 (9.2)	75	70 to 80	70.0 (8.8)	71	65–76	< 0.001
Sex: male (*n*, %)	37 (39%)			62 (48%)			0.21
BMI (kg/m^2^)	27.3 (5.4)	26.5	24.5 to 30.0	26.3 (5.3)	26.5	22.9–29.0	
FEV1%	76.6 (26.3)	77	60 to 90	88.0 (22.4)	91	73–101	< 0.001
DLCO %	71.0 (22.1)	71	57 to 82	83.4 (21.1)	82	68–97	< 0.001
CCI	2.1 (1.3)	2	1 to 3	1.2 (1)	1	0–2	< 0.001
PS > 1 (*n*, %)	54 (57%)			21 (16%)			< 0.001
CAD (*n*, %)	32 (34%)			9 (6.9%)			< 0.001
CVD (*n*, %)	12 (13%)			4 (3.1%)			0.008
CKD (*n*, %)	7 (7.4%)			1 (0.8%)			0.011
Diabetes (*n*, %)	22 (23%)			10 (7.6%)			0.001
COPD (*n*, %)	44 (46%)			38 (29%)			0.009
All complications (*n*, %)	85 (89%)			95 (73%)			0.002
Minor complications (Grades 1–2)	64 (67%)			68 (53%)			0.028
Major complications (Grades 3–5)	21 (22%)			27 (21%)			0.81
Treatment related deaths within 90 days (*n*, %)	3 (3%)			7 (5%)			0.52
Baseline QLQ-C-30 Global health (Mean (SD), (*n* completers))	53.8 (23.6), (*n* = 74)			71.2 (16.7), (*n* = 70)			< 0.001
LC-13 Dyspnoea (Mean (SD), (*n* completers))	36.9 (26.9), (*n* = 69)			20.8 (22.1), (*n* = 63)			< 0.001
DSE total score (mean (SD), (*n* completers))	79.5 (23.1), (*n* = 73)			83.6 (22.9), (*n* = 85)			0.09

Results are expressed as means and standard deviations for numeric variables and as counts and percentages for categoric variables

*SABR* stereotactic ablative radiotherapy, *IQR* interquartile range, *BMI* body mass index, *PS* Eastern Cooperative Oncology Group (ECOG) performance score, *FEV1* forced expiratory volume in 1 s, *DCLO* carbon monoxide lung diffusion capacity, *CCI* Charlson comorbidity index, *CAD* coronary artery disease, *CVD* cerebrovascular disease, *CKD* chronic kidney disease, *COPD* chronic obstructive pulmonary disease, *DSE* Decision self-efficacy, *EORTC QLQ C-30 GH* Global Health score of the EORTC QLQ C-30 questionnaire: 1–100 score with higher score representing better quality of life, *LC-13 Dyspnoea* Dyspnoea score of the EORTC Lung Cancer 13 Module: 1–100 score with higher score representing worse degree of symptoms

Clinical outcomes analysis demonstrated that patients treated with SABR experienced a higher proportion of Grade 1–2 complications, as defined by the Common Terminology Criteria for Adverse Events (CTCAE) v3.0 (Trotti et al. 2003) resulting in a higher rate of complications overall (Table 2).

### Patients’ satisfaction results

The patient satisfaction questionnaire, administered at 6 weeks, was completed by 60 SABR (66.6%) and 74 VATS patients (62.7%). We did not find any significant differences between the two groups in any of the PSQ-18 questionnaire subscales. In all scales, patients reported a moderate level of satisfaction with the care provided (Table 3).

**Table 3:** Patient satisfaction results according to the treatment

PSQ-18 SUBSCALES^a^	SABR (*n* = 60)	Surgery (*n* = 74)	*p* value
General satisfaction (mean, SD)	3.82 (1)	3.89 (1)	0.6
Technical quality	3.88 (0.8)	3.95 (0.6)	0.81
Interpersonal manner	4.05 (0.99)	4.37 (0.6)	0.11
Communication	3.95 (0.93)	3.96 (0.81)	0.7
Financial aspects	4 (0.98)	4.08 (0.92)	0.7
Time spent with doctor	3.78 (0.9)	3.85 (0.9)	0.55
Accessibility and convenience	3.44 (0.7)	3.47 (0.8)	0.62

The continuous variables are categorized and split at the mean value

^a^All items are scored (1–5) so that high scores reflect satisfaction with medical care

*SABR* stereotactic ablative radiotherapy, *SD* standard deviation

The comparison between known groups (IMD domains, age, PS, gender and complications) in terms of general satisfaction showed that patients living in more deprived areas (higher IMD scores) were more satisfied. Patients experiencing minor and major complications were also more satisfied 6 weeks after treatment (Table 4). The continuous variables are categorized and split at the mean value.

**Table 4:** General patient satisfaction scores difference in known groups

Group	IMD^a^ > 4.6 (most deprived)	IMD < 4.6 (least deprived)	*p* value
	4.02 (0.9)	3.66 (1)	0.02*

Group	Age > 72	Age < 72	*p* value
	3.8 (1)	3.89 (0.9)	0.85

Group	PS^b^ > 1	PS < 1	*p* value
	3.7 (1)	3.9 (0.9)	0.24

Group	Major complications	No major complications	*p* value
	4.06 (0.97)	3.72 (1)	0.04*

Group	Minor complications	No minor complications	*p* value
	4.06 (0.99)	3.72 (1)	0.03*

Group	Female	Male	*p* value
	3.83 (1)	3.88 (1)	0.64

Results are expressed as mean (SD)

*Statistical significance

^a^IMD: Index of multiple deprivation score 1–10. For all domains of the IMD, a greater score corresponds to greater deprivation

^b^Eastern Cooperative Oncology Group (ECOG) Performance Score

### Patient satisfaction model

The univariate analysis of patient satisfaction was carried out on 134 patients (60 SABR + 74 VATS) who completed the PSQ-18. Results of the regression are shown in Table 5. Multivariable regression analysis showed that the only pre-treatment factors that remained independently associated with patient satisfaction were decision-making self-efficacy and age.

**Table 5:** Generalised linear regression of patient satisfaction by preoperative factors (*N* = 134)

Characteristic	Patient satisfaction (*N* = 134)		Unadjusted standardised coefficient (beta)	CI	*p* value	Adjusted standardised coefficient (beta)	CI	*p* value
	No. or mean	% or SD						
Gender								
Female	76	56.7	0			0		
Male	58	43.3	− 0.02	− 0.19 to 0.15	0.80	− 0.05	− 0.22 to 0.12	0.53
Age (years)								
≤ 65	25	18.7	− 0.62	− 0.64 to 0.02	0.003*	− 0.47	− 0.89 to − 0.05	0.03*
65–75	58	43.3	0	− 1.03 to − 0.21		0		
75+	51	38.1	− 0.31	− 0.64 to 0.02	0.07	− 0.21	− 0.56 to 0.13	0.22
Treatment								
VATS	74	55.2	0			0		
SABR	60	44.8	− 0.03	− 0.20 to 0.14	0.71	0.001	− 0.17 to 0.17	0.99
DSE								
High > 90	62	46.3	0			0		
Low < 90	52	38.8	− 0.44	0.70 to − 0.18	0.001*	− 0.38	− 0.64 to − 0.11	0.005*
Not known	20	14.9	− 0.15	− 0.42 to 0.20	0.41	− 0.14	− 0.49 to 0.21	0.43
Index of multiple deprivation								
3, 4 or 5 (most deprived)	73	54.5	0			0		
1 or 2 (least deprived)	61	45.5	− 0.18	− 0.34 to − 0.01	0.04	− 0.10	− 0.27 to 0.07	0.25
FEV1	1.9	0.70			0.62			
CCI	1.5	1.15			0.56			
ECOG performance								
≤ 1	88	65.7						
> 1	38	28.4			0.25			
Not known	8	6.0			0.50			

*SD* standard deviation, *CI* confidence interval, *VATS* video-assisted thoracoscopic surgery, *SABR* stereotactic ablative radiotherapy, *DSE* decision self-efficacy, *FEV1* forced expiratory volume in 1 s, *CCI* Charlson comorbidity index

^*^Statistical significance at < 0.01

## Discussion

Patient satisfaction results showed no difference between the two treatments for early-stage NSCLC. However, patients who experienced minor or major complications reported higher scores of patient satisfaction. We also demonstrated in our cohort that patients living in the more deprived areas reported higher satisfaction with care, although this association was no longer present in the regression analysis. Further, patients who felt more able to be involved in their treatment decision-making and patients who were older were more satisfied with care.

That patient satisfaction measure offers health services something different to clinical indicators, so is useful in helping services to identify ways to improve patient care. The fact that no association was detected between clinical indicators and patient satisfaction probably does not reflect the effect of the disease and treatment but rather provides information about the service.

Our results are discordant to the work by Barlesi et al. who found that the absence of postoperative complications in similar thoracic surgery patients, was the only quality index that showed weak but significant positive correlation with an index rating the satisfaction with the structure (Barlesi et al. 2005).

Furthermore, results from this study regarding patient satisfaction and post-treatment morbidity also differ from those we have previously found in lung resection patients (Pompili et al. 2015) and in other studies using the European Organisation for Research and Treatment of Cancer (EORTC) IN-PATSAT32 (Bredart et al. 2005). The EORTC In-PATSAT32 is a multi-dimensional questionnaire, adapted to measure patient satisfaction related to physician and hospital staff, as well as aspects of organisation of care and services (Bredart et al. 2005). It demonstrates reliability having been used in previous research (Pompili et al. 2013). It could be argued that some of the dissatisfaction caused by adverse events after surgery may not be alleviated by clear communication or the intensive care that complicated patients require. However, in this previous study, we used a different questionnaire which was administered at discharge. These factors may have influenced these divergent results. The two groups included in the current analysis were heterogenous and the SABR population are day-cases and were, therefore, unable to rate satisfaction with the system and staff to the same extent that patients would be able to during an inpatient journey.

More recently, a US study found that patient satisfaction scores amongst patients undergoing surgery for lung cancer were not significantly affected by postoperative complications. Satisfaction in the areas of communication with doctors and nurses, however, decreased significantly with increasing length of stay suggesting an important role on enhancing provider communication skills (Singer et al. 2019).

However, it is not easy to put these results in the context of studies exploring possible correlation between socio-economic level and patient satisfaction. Comparability across studies is hampered by heterogeneous reporting and differences in participant demographics. Systematic reviews show that socio-economic factors influence the use of health care services and subsequent survival rates in lung cancer patients (Forrest et al. 2013; Finke et al. 2018). In previous reports from our centre, there did not appear to be a significant relationship between socio-economic status and stage, performance status or outcome from lung cancer (Cheyne et al. 2013). Results from the UK National Cancer Patient Experience Survey (2012) showed that the significant differences that exist among IMD and satisfaction of care are not unidimensional (Department of Health and Social Care 2012). However, there was a certain degree of consistency in the kind of questions that were less highly ranked by patients in the most deprived areas as identified by the IMD quintile, with most of the items relating to the providing and understanding of information. There may be some other factor in our population which resulted in the most socio-economically deprived patients being more satisfied with care. On the other hand, the more affluent patients have lower satisfaction, as they may have higher expectations and demands, having more access to external resources or private healthcare, which eventually may be translated into more information and confidence in seeking good care. Future analysis comparing our results with more up-to-date data from the 2017 National Cancer Patient Experience Survey may help to explore these differences and help commissioners to reduce inequalities.

In our study group, there was a positive association between preoperative DSE scores and post-treatment satisfaction, supporting our initial hypothesis. These results may be affected by the 6-week time interval selected for data collection. Participant attrition was greater at this point, especially in the VATS group, but the decision was made to assess satisfaction closer to hospital recovery to allow for a better distinction among elements of satisfaction and response variability (Kane et al. 1997).

Patients living in least deprived areas reported lower patient satisfaction and may in fact be DSE driven. The deprived group has a higher proportion of those with high DSE (61%) compared to the affluent group (46%).

Our data suggest that patients reported lower patient satisfaction if they also have low DSE whether they are in the affluent or the deprived group. The difference in DSE may be more conflict from the researched, informed affluent group (borderline sig *p* = 0.1) and could suggest that the association between IMD and satisfaction is no longer significant in the multivariable model because the association with DSE is stronger.

Decision self-efficacy is not a measure of quality of the decision-making process, but rather measures the involvement of the patient in key decisions relating to treatment. But it has highlighted for the first time, the importance of patient attitudes surrounding treatment choices. Understanding the role of post-treatment patient satisfaction and its association with decision-making factors may provide important information for clinical practice and future research.

## Limitations

This cohort study was designed to explore factors associated with satisfaction and illustrate that patient satisfaction measures have an independent part to play in informing service quality apart from indicators of clinical and treatment effectiveness. However, it was not a randomised control trial of an intervention designed to improve care, so although we can say decision self-efficacy and patient satisfaction are associated, we cannot say why. Prior evidence would suggest that interventions designed to enhance patient involvement in care are likely to increase decision self-efficacy and patient satisfaction with care, for example those involving patient decision aid interventions (Stacey et al. 2017). Although patient-reported measures are essential in understanding patient perspective in care, there are several limitations such as low response rates of questionnaires. In additional, patient satisfaction measures may be subject to social desirability bias in reporting, especially if care is continuing in a service before treatment is completed. Furthermore, the PSQ-18 questionnaire has been designed to assess generic satisfaction and is not specific to either inpatient or outpatient care. This questionnaire was chosen instead of the EORTC PATSAT32, as SABR is an outpatient treatment. We cannot rule out the possibility that this questionnaire may not be capturing the same domains in these two different groups.

## Conclusions

Patient satisfaction measures are important in service improvement as independent of the patient perception of the health problem and burden of symptoms and impact of treatment. It seems likely that supporting patient involvement in treatment decision-making is associated with satisfaction, independent of the treatment type. Socio-demographic factors did not affect reported satisfaction but may have influenced the efficacy in making the decision about treatment for early-stage NSCLC. The understanding of the clinical decision-process from patients deserves more investigation to improve patient-reported satisfaction with care for early-stage NSCLC.

## Data Availability

No data from this study is available for sharing.

## Abbreviations

MIS: Minimally invasive surgery
NICE: National Institute for Clinical Excellence
NIHR: National Institute for Health Research
NSCLC: Non-small cell lung cancer
PROs: Patient-reported outcomes
PROMs: Patient-reported outcome measures
SABR: Stereotactic ablative radiotherapy
VATS: Video-assisted thoracoscopic surgery
